# Impact of Wildfire Smoke on Adverse Pregnancy Outcomes in Colorado, 2007–2015

**DOI:** 10.3390/ijerph16193720

**Published:** 2019-10-02

**Authors:** Mona Abdo, Isabella Ward, Katelyn O’Dell, Bonne Ford, Jeffrey R. Pierce, Emily V. Fischer, James L. Crooks

**Affiliations:** 1 Department of Epidemiology, Colorado School of Public Health, Aurora, CO 80045, USA; mona.abdo@ucdenver.edu; 2 Swedish Medical Center, Englewood, CO 80113, USA; ward.isabella@gmail.com; 3 Department of Atmospheric Science, Colorado State University, Fort Collins, CO 80523, USA; katelyn.odell@colostate.edu (K.O.); Bonne.Ford@colostate.edu (B.F.); Jeffrey.Pierce@colostate.edu (J.R.P.); evf@rams.colostate.edu (E.V.F.); 4 Division of Biostatistics and Bioinformatics and Department of Biomedical Research, National Jewish Health, Denver, CO 80206, USA

**Keywords:** wildfire smoke, PM_2.5_, Colorado, pre-term birth, birth weight, gestational diabetes, gestational hypertension, small for gestational age, assisted ventilation, NICU admission

## Abstract

Colorado is regularly impacted by long-range transport of wildfire smoke from upwind regions. This smoke is a major source of ambient PM_2.5_. Maternal exposure to total PM_2.5_ during pregnancy has been linked to decreased birth weight and other adverse outcomes, although the impact of wildfire smoke contribution has only recently been investigated. The objective of this study was to estimate associations between adverse pregnancy outcomes and ambient wildfire smoke PM_2.5_. Wildfire smoke PM_2.5_ exposures were estimated using a previously published method incorporating ground-based monitors and remote sensing data. Logistic regression models stratified by ZIP code and mixed models with random intercept by ZIP code were used to test for associations. The primary outcomes of interest were preterm birth and birth weight. Secondary outcomes included gestational hypertension, gestational diabetes, neonatal intensive care unit admission, assisted ventilation, small for gestational age, and low birth weight. Exposure to wildfire smoke PM_2.5_ over the full gestation and during the second trimester were positively associated with pre-term birth (OR = 1.076 (μg/m^3^)^−1^ [95% CI = 1.016, 1.139; *p* = 0.013] and 1.132 (μg/m^3^)^−1^ [95% CI = 1.088, 1.178]; *p* < 0.0001, respectively), while exposure during the first trimester was associated with decreased birth weight (−5.7 g/(μg/m^3^) [95% CI: −11.1, −0.4; *p* = 0.036]). Secondary outcomes were mixed.

## 1. Introduction

Areas affected by wildfire smoke experience elevated levels of air pollutants, including particulate matter (PM), aromatic hydrocarbons, carbon monoxide, aldehydes and a large suite of other volatile organic compounds. These pollutants have each been associated with acute and/or chronic adverse pregnancy outcomes, including fetal malformation, fetal death, and low birth weight [1,2], though not necessarily in the context of wildfire smoke. Several studies have been conducted examining the impact of single wildfire smoke events on birth weight in California and Australia [3,4], but the aggregate impact of wildfire smoke over a multi-year fire record on adverse birth outcomes has not yet been examined. Chronic maternal exposure to ambient PM and indoor biomass smoke during pregnancy have been linked to decreased infant birth weight. In a study conducted in Connecticut and Massachusetts over six years (from 2000 to 2006), it was concluded that exposure to PM_2.5_ (particulate matter smaller than 2.5 μm in aerometric diameter) during pregnancy (over the entire pregnancy) was associated with low birth weight (LBW) and small for gestational age (SGA) [5]. In a 32-study meta-analysis conducted by Sun et al. [6] done to determine the association between birth weight and exposure to fine particulate matter (PM_2.5_), it was concluded that there was a statistically significant association between birth weight and PM_2.5_ exposure over the entire pregnancy (−1.6 g/(μg/m^3^) [95% CI = −2.7, −0.5]). From the same meta-analysis, it was also concluded that there was a statistically significant association between PM_2.5_ exposure during the entire pregnancy and LBW (OR = 1.009/(μg/m^3^) [95% CI = 1.003, 1.015]). The overall conclusions from the meta-analysis was that PM_2.5_ exposure during pregnancy was associated with low birth weight, and the late stages of the pregnancy were found to be the most critical periods [6]. Holstius et al. [3] showed that after adjusting for infant sex, gestational age at birth, and other factors known to influence birth weight; the exposed infants weighed an average of 6.1 g less at birth than unexposed infants. Infants exposed during the second trimester showed the largest average reduction, at 9.7 g. However, O’Donnell et al. [4] found that male infants born in the highly fire-affected area weighed significantly more on average than male infants born in areas less impacted by wildfire smoke and male infants born in the same areas during non-fire years. Increases in macrosomic infants contributed to the higher average birth weight. There was no significant effect on the female infant weight or gestational age for either gender. In another comprehensive review and meta-analysis conducted by Sapkota et al. [7] to determine the association between particulate matter and adverse birth outcomes, it was concluded from 20 studies that there was a significant association between PM_2.5_ exposure and risk of pre-term births (combined OR = 1.015/(μg/m^3^) (95% CI = 1.014, 1.016)).

Exposure to wildfire smoke has also been linked to many other adverse health outcomes including respiratory disease, cardiovascular disease, and all-cause mortality [8,9,10,11,12,13,14] A systematic search for studies assessing the association between non-occupational exposure to wildfire smoke and physical health impacts concluded that there is (1) consistent evidence for an association between exposure to wildfire smoke and risk of respiratory outcomes and (2) suggestive evidence for an association between exposure to wildfire smoke and risk of cardiovascular outcomes [15]. In another systematic review, it was concluded that there was consistent evidence for associations between wildfire smoke exposure and respiratory health effects and increasing evidence to support an association between wildfire smoke exposure and all-cause mortality [16].

Our work is timely because the area burned by wildfires in the western US and Western Canada has increased in recent decades [17,18]. While results vary, there are now many studies predicting worsened summertime air quality from wildfires in the western US as the region continues to warm in response to greenhouse gas emissions [19,20,21,22,23,24]. Colorado is susceptible to increased wildfires locally. As wildfires and populations increase, more people in Colorado will be exposed to smoke. This was confirmed in a study by Lui et al. [25], in which multiple factors, including wildland-urban interface expansion in the Colorado Front range as well as the changing fire regime, are contributing to wildland-urban interface burn. In addition, given the dominant transport patterns of smoke, increased fire activity in Pacific the Northwest and California will likely increase smoke-exposure in Colorado as well. The state routinely receives smoke from these fire-prone regions [26,27]. Understanding the association between the pollutants that are released from the fires and birth outcomes is of great public health importance, given the expected continued increases in population and smoke exposure in the state.

This study aims to characterize the association between wildfire smoke PM_2.5_ exposure and birth outcomes by trimester. Characterizing this relationship will help public health professionals develop more targeted interventions, such as recommendations and guidelines, aimed at reducing the impact on babies and mothers. Furthermore, and while this is not our primary goal, our results could help generate hypotheses about which cellular or molecular mechanisms are impacted by maternal exposure to ambient air pollution, thereby driving adverse clinical outcomes.

## 2. Materials and Methods

### 2.1. Population

Birth outcome and individual-level covariate data were extracted from the Colorado Vital Records Registry, maintained by the Colorado Department of Public Health and Environment (CDPHE). This data set aggregates elements from all birth certificates (the Colorado Standard Certificate of Live Birth) issued in Colorado. The data set was provided to the authors under a data use agreement with CDPHE. This study was deemed not to constitute human research as the data are publicly available, de-identified, and did not involve investigator contact with study subjects. These data were supplied by the Center for Health and Environmental Data, Registries and Vital Statistics Branch, of the CDPHE, which specifically disclaims responsibility for any analyses, interpretations, or conclusions it has not provided. Privacy considerations limited the birth certificate data set to report only the year and month of birth, not the exact birth date. The impact of this temporal uncertainty on exposure characterization is described below.

There were a total of 589,992 birth records, of which 535,895 met the inclusion criteria. Inclusion criteria included singleton births between the years of 2007 and 2015 in Colorado with estimated gestational age between 30 and 42 weeks. Full maternal address was not included in the data set, so exposures were linked to mothers by maternal residence ZIP code. All Colorado ZIP codes had at least one day during the years of the study with a non-zero ambient wildfire smoke PM_2.5_ concentration. Therefore, all Colorado ZIP codes with at least one single birth during the study period were included in the analyses. Descriptive summaries of the birth cohort are given in Table 1.

The primary outcomes of interest in this study were pre-term birth and birth weight. Births with estimated gestational age of 37 weeks or less were categorized as pre-term while births with estimated gestational age greater than 37 weeks were categorized as full-term. Maps of the number of births by ZIP code, the percentage of pre-term births by ZIP code, and the mean birth weight by ZIP code are shown in Supplemental Figures S1–S3, respectively. While the percentage of pre-term births does not display any visible geographic patterns, ZIP codes at high altitude tend to have lower average birth weights.

Secondary outcomes of interest included gestational diabetes, gestational hypertension, neonatal intensive care unit (NICU) admission, assisted ventilation following delivery, low birth weight (LBW), and small for gestational age (SGA). For the gestational diabetes analysis, mothers who had pre-pregnancy diabetes were excluded. Similarly, for the gestational hypertension analysis, mothers who had pre-pregnancy hypertension were excluded. Births with weight below the 10th percentile for the gestational age were considered as small for gestational age. Infants weighing less than 2500 g were categorized as LBW while infants weighing 2500 g or more were categorized as normal birth weight. Summary statistics for all outcomes are given in Supplemental Table S1.

Due to low incidence and the limited temporal precision in our exposures, we did not study outcomes associated with conception itself, such as chromosomal disorders, or outcomes with narrow developmental windows, such as cleft lip/palate, gastroschisis, or neural tube defects, despite the availability of this data.

Birth certificate covariates considered for potential inclusion in the models included: mother’s education categorized as either less than high-school, high-school, bachelors, or more than bachelor’s degree; graduated index (which provides information on prenatal care as is commonly shortened to ‘gindex’); birth year; birth calendar month; alcohol consumption during pregnancy defined as either yes (drinking during any trimester) or no; smoking during pregnancy defined as either yes (smoking during any trimester) or no; income categorized as <$25,000, $25,000, $49,999, $50,000–$74,999, or $75,000+; asthma defined as either yes or no, and number of prenatal visits categorized as <10 or 10+. We collapsed maternal race/ethnicity into three categories (Hispanic, non-Hispanic White, or non-Hispanic non-White) because the birth certificate allowed for several hundred self-reported race/ethnicity combinations, only a few of which had a substantial number of births. We trichotomized maternal age according to the standard age categories used in obstetrics as indicators of risk (less than 18, 18–35, or greater than 35 years). All the other variables were self-reported or physician-reported on the birth certificate in the categories given.

### 2.2. Exposure Characterization

**Figure 1:**
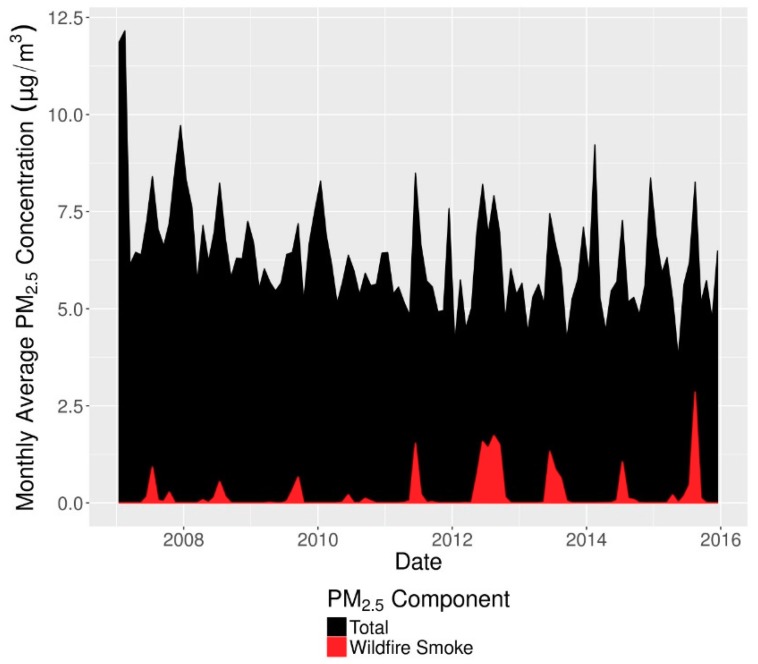
Time series of monthly wildfire (red) and total PM_2.5_ (black) concentrations in Colorado. Values are averages of ZIP code-level concentrations weighted by number of births.

Wildfire smoke PM_2.5_ and non-smoke PM_2.5_ were jointly characterized using a previously published method [28] that has been used in a prior health study [29]. This method combines NOAA’s satellite imagery-based Hazard Mapping System [30,31,32] to determine daily smoke plume extent with spatial interpolation of ground-based PM_2.5_ monitor values downloaded from the US EPA Air Quality System (AQS). Daily concentrations of both components over a 15 × 15 km grid covering the contiguous US were then matched to ZIP codes using the mean of the concentrations in each grid cell overlapping the ZIP code, weighted by the population in each grid cell and the areal overlap between the grid cell and ZIP code. Monthly time series of total PM_2.5_ and wildfire smoke PM_2.5_ in Colorado are shown in Figure 1. Wildfire smoke PM_2.5_ concentrations increased both in absolute terms and relative to total PM_2.5_, which itself decreased during our study period. In the latter years of the study wildfire smoke became a major contributor to PM_2.5_ peaks during summer months.

**Figure 2:**
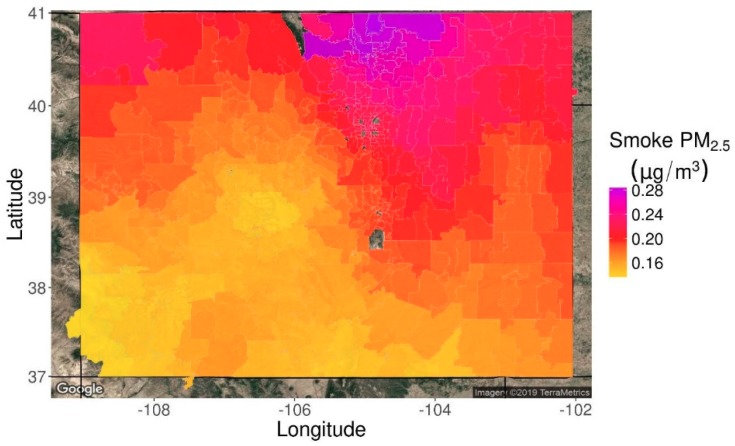
Map of mean wildfire smoke exposure density by ZIP code averaged over the full nine-year dataset. ZIP codes without recorded births are omitted.

A map of ZIP code-specific daily wildfire smoke PM_2.5_ concentrations averaged over the study period is shown in Figure 2. The northern portion of the Front Range including Fort Collins experienced the highest wildfire smoke PM_2.5_ concentrations in the state. The Denver metropolitan region and the northwest and northeast corners also experienced high concentrations relative to the southern half of the state. A significant fraction of smoke in Colorado comes from smoke transported from the US Pacific Northwest and Western Canada [26]. A common route for this transported smoke flows around the Rocky Mountains, through the plains of eastern Wyoming into the northern Front Range region of Colorado (the most smoke impacted portions of Figure 2). From the northern Front Range, the smoke disperses either east or south following topography and wind patterns.

Other exposure covariates included in the statistical models were ambient temperature, PM_10_, and ozone. PM_10_ was included instead of coarse PM because PM_10_ is directly monitored, and not all of the sites with PM_10_ monitors also have PM_2.5_ monitors, making it difficult to estimate the spatial distribution of coarse PM. However, PM_2.5_ is a small contributor to PM_10_ mass, and concentrations of the two are not strongly correlated (in our dataset PM_10_ has correlations of 0.44, 0.15, and 0.26, respectively, with wildfire smoke PM_2.5_, non-smoke PM_2.5_, and total PM_2.5_). ZIP code-specific daily values of these variables were computed, as in [29], by taking the median of all reported values from monitors falling within the ZIP code boundary or within 20 km of its centroid. In contrast to the wildfire smoke and non-smoke PM_2.5_ exposure characterization above, temperature, PM_10_, and ozone values were not spatially interpolated due to concerns about interpolation accuracy given the sparseness of their monitor networks. Because these variables were not interpolated, grid cell population weighting was not performed. A 20 km buffer distance was chosen as the default as a compromise between accuracy and power. A smaller buffer implies more accurate characterization of population exposure, but at the cost of more ZIP codes with missing exposure data and lower power. A larger buffer implies less accurate exposure characterization but fewer ZIP codes with incomplete data and thus higher power. The impact of varying this buffer distance is explored in sensitivity analyses.

Because the calendar month of birth was already included in the gestational outcome models and indirectly accounted for seasonal changes in temperature, the temperature variable was normalized to the time of year by subtracting the monthly mean temperature. This variable is referred to as “Temperature Deviation” in the tables below. Summary statistics for the exposure variables are given in Table 2.

Because the exact birth date was unavailable and because the gestational age was estimated at a weekly resolution, there was uncertainty regarding the exact start and end dates of each gestation and of individual trimesters. This uncertainty precluded analyzing the gestational period at a more refined temporal resolution than trimester. Indeed, models run using monthly estimates of exposure yielded results that were not robust under sensitivity analysis as a consequence of low exposure accuracy and over-parameterization.

Trimester-specific exposures were calculated as follows. First, our estimate for the earliest conception date was determined by taking the first day of the birth month and subtracting the gestational age in weeks and an additional six days. Our estimate for the latest conception date was determined by taking the last day of the birth month and subtracting the gestational age in weeks. These were then defined as the earliest and latest possible start dates for the first trimester. The earliest end date of the first trimester was calculated by adding 12 weeks to the earliest start date. The latest end date of the first trimester was calculated by adding 12 weeks and six days to the latest start date. The earliest and latest start/end dates for other trimesters were calculated similarly, with the second trimester defined to end at 27 weeks and six days. 

Thus, for each trimester, three adjacent intervals were defined: first, between the earliest and latest start date; second, between the latest start and earliest end date; and third, between the earliest and latest end date. Finally, the average exposure over a trimester was calculated by taking a weighted mean of daily ZIP code-resolution values over the days in these three intervals. The values in the second interval (where there is most certainty) were all given a weight of 1, while in the first and third intervals the weights decreased linearly to zero toward the earliest start and latest end date. Thus, the trimester exposure estimates were most strongly influenced by values on days that were most certain to fall in the true trimester interval and less strongly influenced by values on days that were less certain to fall in the true trimester interval.

### 2.3. Statistical Analyses

Characteristics of the cohort were summarized using descriptive statistics to determine if there were any differences between the groups (pre-term versus full-term) in the cohort that might lead to confounding. Means and standard deviations were determined for the continuous variables, and frequency and percentages were determined for the categorical variables for the overall cohort and for the main outcome of interest for pre-term versus full-term births. Multi-collinearity was evaluated between income, education, race/ethnicity, smoking and drinking using the variance inflation factor (VIF). All VIFs were less than 3.4, which is below the standard threshold (VIF > 10) for dropping or combining variables. Therefore, we retained each maternal variable (income, education, race/ethnicity, smoking and drinking) in the models.

For the pre-term birth outcome, logistic regression models were developed to estimate associations between lagged wildfire smoke PM_2.5_ exposures and pre-term birth. The model included a separate strata (intercept) for each residential ZIP code and accounted for wildfire smoke PM_2.5_ (three trimesters), ozone (three trimesters), non-wildfire PM_2.5_ (three trimesters), PM_10_ (three trimesters), normalized temperature (three trimesters), calendar month of birth, birth year, maternal age, race/ethnicity, education, and income, maternal smoking during pregnancy, maternal alcohol consumption during pregnancy, maternal asthma, and gindex.

For continuous birth weight, mixed effects models with a random intercept by ZIP code were used to estimate associations with wildfire smoke PM_2.5_. This model used the same predictor variables as in the preterm birth models but with additional control for gestational age using a nonlinear spline fit with 5 degrees of freedom.

For the secondary outcomes of interest (NICU, gestational diabetes, gestational hypertension, assisted ventilation at time of delivery, SGA, and LBW), logistic models were used to estimate associations with wildfire smoke PM_2.5_. These secondary models accounted for all confounders accounted for in the preterm birth model as well as a nonlinear control for gestational age as used in the birth weight model. For LBW, infants were categorized into two birth weight categories (low birth weight (<2500 g) or normal birth weight (≥2500 g)).

Estimates of association over the full gestational period were calculated in two ways. First, the mean effect over all three trimesters was computed using the contrast statement in SAS with each trimester effect weighted by 1/3. Second, models were run using full gestation values of the air pollutants and temperature rather than trimester-specific values.

Sensitivity analyses were also conducted to determine if there were any changes in the overall results for the primary outcomes under different data processing and confounder choices. In total. eight sensitivity analyses were performed for each of the two main outcomes: (1) the main model without PM_10_; (2) the main model without ozone, PM_10_, or non-wildfire PM_2.5_; (3) the main model with infant gender included; (4) the main model without temperature; (5) the main model using a 5-km buffer distance; (6) the main model using a 10-km buffer; (7) the main model using a 50-km buffer; and (8) the main model with ZIP code-level wildfire smoke and non-wildfire PM_2.5_ values computed without population weighting.

All data were analyzed using SAS 9.4 (SAS, Cary, NC, USA), and an alpha level of 0.05 was used to determine significance in all the statistical tests and confidence intervals. Logistic analyses were performed using PROC Logistic while birth weight was analyzed using PROC hpmixed.

## 3. Results

### 3.1. Descriptive Analysis

Table 1 presents descriptive statistics for our study population. The majority of the mothers were between ages 18 and 35 (85.60%), did not have asthma (95.41%), did not drink during their pregnancy (98.93%), did not smoke during their pregnancy (92.42%), were Non-Hispanic White (63.61%), and had more than 10 prenatal visits (60.19%). Roughly half had a high-school degree (49.08%). Among mothers who had a preterm birth, the proportion of mothers who had less than 10 prenatal visits was higher (51.32% versus 48.68%), while among mothers who did not have a preterm birth, the proportion of mothers who had 10 or more prenatal visits was higher (62.06% versus 37.94%). Other characteristics were similar between mothers who had a pre-term birth and mothers who had a full-term birth.

Table 2 presents descriptive statistics for the main exposure and continuous covariates. Trimester-average wildfire smoke PM_2.5_ concentrations were generally low, with a mean of 0.2 μg/m^3^ and a maximum of 4.5 μg/m^3^. This reflects the sporadic nature of smoke events in Colorado. Concentrations of non-smoke PM_2.5_ were generally higher, though normally well below the US EPA primary annual standard of 12 μg/m^3^.

### 3.2. Primary Endpoints

As shown in Table 3, exposure to wildfire smoke PM_2.5_ during the second trimester was significantly associated with preterm birth (OR = 1.132 (μg/m^3^)^−1^ [95% CI = 1.088, 1.178]; *p* < 0.0001) after adjusting for co-exposures and known risk factors for preterm births. Other trimesters did not yield significant associations. However, exposure over the entire pregnancy was significantly associated with preterm birth (OR = 1.076 (μg/m^3^)^−1^ [95% CI = 1.016, 1.139; *p* = 0.013]).

Furthermore, as shown in Table 4, each additional 1 μg/m^3^ increase in trimester-average wildfire smoke PM_2.5_ exposure during the first trimester was associated with −5.7 g [95% CI: −11.1, −0.4; *p* = 0.036] change in birth weight. Wildfire smoke PM_2.5_ was not associated with birth weight in any other trimester.

### 3.3. Secondary Endpoints

Associations between wildfire smoke PM_2.5_ exposure and secondary outcomes, adjusted for confounders, are shown in Table 5, Table 6 and Table 7. Among the maternal outcomes, there was a significant positive association between exposure to wildfire smoke and gestational diabetes during the first trimester (OR = 1.144 (μg/m^3^)^−1^ [95% CI = 1.064, 1.230; *p* = 0.0003]) as well as over the entire pregnancy (OR = 1.151 (μg/m^3^)^−1^ [95% CI = 1.034, 1.281; *p* = 0.010]). There was also a significant positive association between exposure and gestational hypertension during the first trimester (OR = 1.148 (μg/m^3^)^−1^ [95% CI = 1.071, 1.231; *p* = 0.0001]), second trimester (OR = 1.124 (μg/m^3^)^−1^ [95% CI = 1.044, 1.211; *p* = 0.0020]), and over the entire pregnancy (OR = 1.204 (μg/m^3^)^−1^ [95% CI = 1.083, 1.339; *p* = 0.0006]). 

Associations with NICU admission and assisted ventilation were contrary to our expectation. Exposure over the second trimester and entire pregnancy was negatively associated with NICU admission (OR = 0.927 (μg/m^3^)^−1^ [95% CI = 0.871, 0.988; *p* = 0.019] and 0.799 (μg/m^3^)^−1^ [95% = 0.730,0.874; *p* < 0.0001], respectively), while exposure over the first trimester, second trimester, and the entire pregnancy were negatively associated with assisted ventilation (OR = 0.772 (μg/m^3^)^−1^ [95% CI = 0.712, 0.836; *p* < 0.0001], 0.916 (μg/m^3^)^−1^ [95% CI = 0843, 0.996; *p* = 0.041], and 0.583 (μg/m^3^)^−1^ [95% CI = 0.515, 0.659; *p* < 0.0001], respectively).

Exposure was not associated with LBW in any trimester and associated with SGA only in the first trimester, and then only weakly (OR = 1.060 (μg/m^3^)^−1^ [95% CI = 1.001, 1.124; *p* = 0.047]).

### 3.4. Sensitivity Analyses

For preterm birth (Supplemental Table S2), all sensitivity models showed a positive association in the second trimester, with an odds ratio point estimate ranging from 1.101 (μg/m^3^)^−1^ (50 km buffer) to 1.225 (μg/m^3^)^−1^ (5-km buffer). Furthermore, the mean association over all trimesters remained significantly positive in all sensitivity models—except when using the 5-km buffer. However, the positive association we found for the full gestation exposure was not as robust, only remaining significant at the *p* < 0.05 level in five of the eight models.

For continuous birth weight (Supplemental Table S3), the association with first trimester exposure found in our main model was not robust to changes in model specification or our processing pipeline. Only under two of the eight sensitivity models (the models without population weighting and with infant gender) did the association remain significant at the *p* < 0.05 level. However, the effect magnitude was more consistent; under seven of the eight models the point estimate fell between −7 and −3 g/(μg/m^3^).

## 4. Discussion

Exposure to wildfire smoke PM_2.5_ during the second trimester was positively associated with preterm birth. Specifically, each 1 μg/m^3^ increase in trimester-average wildfire smoke PM_2.5_ over the second trimester was associated with a 13.2% increase in the odds of preterm birth. For a woman whose second trimester corresponds to peak wildfire season (June, July, and August) and whose exposure equals the Colorado average for the season (0.548 μg/m^3^), this translates to a 7.0% increased odds of pre-term birth. This result was robust to changes to the statistical model and the data processing pipeline. Positive associations with preterm birth were also found after averaging the three trimester-specific associations and when modeling exposure over the full gestational period, though the latter result was not consistently replicated in all sensitivity analyses.

Among secondary end points, the maternal outcomes (gestational diabetes and hypertension) were positively associated with wildfire smoke PM_2.5_, though the strongest results were found when this exposure took place in the first trimester of pregnancy. Each additional μg/m^3^ increase in trimester average exposure was associated with a 14.4% increase in gestational diabetes and a 14.8% increase in gestational hypertension. However, among infant outcomes, results for SGA and LBW were null or weak, while results for NICU admission and assisted ventilation were contrary to our expectation.

Results for birth weight were similar to Holstius et al. [3], in which wildfire exposure resulted in decreased birth weight. While Holstius et al. had a larger sample size (n = 886,034) and characterized exposure differently, both studies found a negative association between wildfire smoke exposure and birth weight, though in different trimesters (−5.7 g/(μg/m^3^) in the first trimester and -9.7 g in the second trimester for the present work and Holstius et al., respectively). However, the present work used a continuous exposure variable while Holstius et al. dichotomized pregnancies into exposed/not exposed categories based on reports of fires from the California Department of Forestry and Fire Protection, making comparisons of effect magnitude problematic. Their paper also had different exclusion criteria than our study; they excluded preterm births and post-term births, while we included preterm births after 30 weeks in our analysis.

While no study has directly tested for differences in birth outcome associations between wildfire smoke PM_2.5_ and total PM_2.5_ or non-smoke PM_2.5_, Sun et al. [6] report a pooled (over 32 studies) estimate for birth weight and total PM_2.5_ of −1.59 g/(μg/m^3^) (95% CI: −2.68, −0.50) over the full gestation, which is similar to our estimate of −2.0 g/(μg/m^3^) for smoke PM_2.5_ over the same period. This similarity suggests non-differential impacts on birth weight. However, we found the strongest associations with birth weight in the first trimester while Sun et al. found the opposite, which may reflect the lack of robustness in our birth weight results.

Prior work on criteria air pollutants and birth outcomes has found increases in preterm birth risk and decreases in birth weight with total PM_2.5_ and PM_10_. Lamichhane et al. [33] found a 2.2 g/(μg/m^3^) decrease in birth weight and a 1.4% (μg/m^3^)^−1^ increase in preterm birth risk over the entire pregnancy. The former is similar in magnitude to our (non-significant) result, while the latter is lower than the 7.6% (μg/m^3^)^−1^ increase that we observed. A study in New York City [34] found a 4.8 g/(μg/m^3^) decrease in birth weight with PM_2.5_ mass, while NO_2_ decreased birth weight by 1.8 g/ppb. Coarse PM has also been found to negatively impact birth weight [35].

Mechanical assisted ventilation provides crucial breathing support to neonates, but prolonged ventilation is a risk factor for bronchopulmonary dysplasia [36]. While our finding of a negative association between wildfire smoke exposure and assisted ventilation after delivery is counter to our original hypothesis, there is limited evidence suggesting that maternal cigarette smoking during pregnancy can lead to accelerated growth in abdomen diameter [37] and reduced risk of respiratory distress syndrome [38]. Thus, there is some support for the theory that adverse pregnancy conditions may lead to accelerated pulmonary maturity. However, a large body of other work has shown detrimental associations between maternal smoking and fetal growth restriction as well as other developmental delays [39,40] including reduced lung function in the week after birth [41,42] and prenatal lung volume [43]. Furthermore, individual ambient air pollutants have also been linked to reductions in fetal abdominal circumference during the first and second trimester [44] and many other adverse pregnancy outcomes [6,7,33,45].

This study had a number of strengths. First, the study sample was relatively large and therefore there was enough power to be able to detect differences in outcome measures. Second, the study investigated a broader range of gestational outcomes than previous studies. Third, the study assessed the impact of wildfire smoke exposure using a spatially and temporally resolved multi-year wildfire exposure record. Fourth, the birth certificate dataset included many individual-specific variables for the assessment of potential confounding.

This study also has a number of limitations. First, many of birth certificate variables were self-reported by the mother, which may have led to misreporting of some confounding variables (e.g., mother’s education, income, drinking during pregnancy, smoking during pregnancy), making it difficult to control for these variables accurately. Second, ambient exposure may not accurately reflect personal exposure, either because of time spent indoors, the use of area (ZIP code) averages rather than residence-specific estimates of the exposure, or changes in residence address over the course of the pregnancy. Third, while our sample size was relatively large (comparable to Holstius et al.), few pregnant women were exposed to significant wildfire smoke for extended periods of time. That being said, showing detrimental effects even at low exposures underscores the importance of potential interventions. Fourth, the birth certificate data did not contain the specific birth dates, but only month and year of birth, and therefore we could not analyze the data at a daily or weekly resolution because we could not accurately assign exposure. In addition, we were not able to conduct the analysis at a monthly resolution because we found that monthly models yielded non-robust results in sensitivity analyses, which suggested that such models were over-parameterized given the amount of data available. The date imprecision may have led to exposure misclassification as some of the exposure assigned to a particular birth or trimester may have occurred either prior to conception, after the birth occurred, or in a different trimester. However, the present work has attempted to account for such uncertainty by weighting the contributions of daily exposures to the trimester and full gestation averages. Future studies may improve the accuracy of the exposure characterization by using birth date rather than birth month, by acquiring mothers’ prior addresses during pregnancy, or by outfitting volunteers with personal or household monitoring devices. Finally, the health effects we ascribe to the effects of wildfire smoke PM_2.5_ may not be due to this pollutant alone but may be due in part to other, unmeasured fire-related air pollutants carried in the same plumes, such as NOx and VOCs, which differ from PM_2.5_ in their health effects and characteristic atmospheric lifetimes. The extent to which wildfire smoke PM_2.5_ tracks with these other pollutants is an active area of investigation by members of our research team.

## 5. Conclusions

This work is the first epidemiologic study to investigate the relationships between ambient wildfire smoke concentrations and pregnancy outcomes in Colorado. We found wildfire smoke PM_2.5_ exposure in the second trimester was positively associated with pre-term birth. Weaker evidence suggested a negative association between first-trimester exposure and birth weight. We also found detrimental effects of wildfire smoke on gestational diabetes and hypertension in the mother, but a seemingly protective association with assisted ventilation of the neonate. It is of great public health importance to develop interventions aimed at reducing pregnant women’s exposure to wildfire smoke. Public health agencies should consider targeting pregnant women when developing wildfire smoke exposure reduction strategies as climate change is expected to increase the frequency and intensity of wildfires in the western US over the next 50 years [19,38,39], which will increase the health burden on expectant mothers and their babies.

## Figures and Tables

**Table 1:** Descriptive statistics.

		Preterm		Birth Weight (g)	
	N	No (%)	Yes (%)	Mean	SD
**Month of Birth**					
January	35,053	29,899 (85.30%)	5154 (14.70%)	3241	495.7
February	33,695	28,874 (85.69%)	4821 (14.31%)	3247.8	489.5
March	37,982	32,631 (85.91%)	5351 (14.09%)	3255	485.8
April	37,200	31,985 (85.98%)	5215 (14.02%)	3257.7	487.6
May	38,396	33,056 (86.09%)	5340 (13.91%)	3257.4	489.9
June	37,820	32,401 (85.67%)	5419 (14.33%)	3256.6	491.3
July	39,411	34,104 (86.53%)	5307 (13.47%)	3252.1	487.7
August	39,581	34,111 (86.18%)	5470 (13.82%)	3256.6	485.8
September	38,606	33,567 (86.95%)	5039 (13.05%)	3256.8	482.8
October	37,372	32,364 (86.60%)	5008 (13.40%)	3257	484.9
November	35,203	30,224 (85.86%)	4979 (14.14%)	3251.6	490.6
December	36,642	31,309 (85.45%)	5333 (14.55%)	3241.3	490.5
**Number of Prenatal Visits**					
<10	177,945	145,903 (81.99%)	32,042 (18.01%)	3194.2	511.5
10+	269,016	238,622 (88.70%)	30,394 (11.30%)	3291.5	468.6
**Birth Year**					
2007	47,648	40,627 (85.27%)	7021 (14.73%)	3242.3	486.8
2008	50,302	42,732 (84.95%)	7570 (15.05%)	3249.2	485.3
2009	50,840	43,487 (85.54%)	7353 (14.46%)	3251	487.5
2010	50,056	43,055 (86.01%)	7001 (13.99%)	3251.4	486.4
2011	49,604	42,964 (86.61%)	6640 (13.39%)	3254.3	488.6
2012	49,029	42,289 (86.25%)	6740 (13.75%)	3256.8	487
2013	49,658	43,140 (86.87%)	6518 (13.13%)	3257	490
2014	49,816	43,234 (86.79%)	6582 (13.21%)	3257.7	490.6
2015	50,008	42,997 (85.98%)	7011 (14.02%)	3254.6	493.8
**Mother’s Race and Ethnicity**					
Hispanic	116,570	99,546 (85.40%)	17,024 (14.60%)	3237.6	489.4
Non-Hispanic Non-White	46,068	38,533 (83.64%)	7535 (16.36%)	3152.7	496.2
Non-Hispanic White	284,323	246,446 (86.68%)	37,877 (13.32%)	3275.1	484.5
**Mother Smoked in Any Trimester**					
No	413,081	356,755 (86.36%)	56,326 (13.64%)	3266.9	485
Yes	33,880	27,770 (81.97%)	6110 (18.03%)	3080.6	497.5
**Income**					
<$15,000	102,329	86,249 (84.29%)	16,080 (15.71%)	3180.9	498.5
$15,000–$24,999	56,126	47,909 (85.36%)	8217 (14.64%)	3227.5	490.3
$25,000–$34,999	44,007	37,472 (85.15%)	6535 (14.85%)	3248.3	493.4
$35,000–$49,999	45,057	38,936 (86.41%)	6121 (13.59%)	3271	483.2
$50,000–$44,999	69,796	60,575 (86.79%)	9221 (13.21%)	3288.8	483.4
$75,000+	129,646	113,384 (87.46%)	16,262 (12.54%)	3296.2	475.1
**Mother Drank Alcohol in Any Trimester**					
No	442,168	380,403 (86.03%)	61,765 (13.97%)	3253.3	488.3
Yes	4793	4122 (86.00%)	671 (14.00%)	3202.9	499.1
**Mother’s Level of Education**					
Less Than High School	64,722	54,898 (84.82%)	9824 (15.18%)	3210.5	501.5
High School	219,354	186,439 (85.00%)	32,915 (15.00%)	3231.7	490.7
Bachelors	107,840	94,834 (87.94%)	13,006 (12.06%)	3300.2	476
Higher than Bachelors	55,045	48,354 (87.84%)	6691 (12.16%)	3293.2	477.9
**Mother has Asthma**					
No	426,436	367,162 (86.10%)	59,274 (13.90%)	3254.7	488
Yes	20,525	17,363 (84.59%)	3162 (15.41%)	3211.5	497
**Mother’s Age**					
<18	8221	6918 (84.15%)	1303 (15.85%)	3134.8	479.8
18–35	382,590	330,139 (86.29%)	52,451 (13.71%)	3253.2	484.3
>35	56,150	47,468 (84.54%)	8682 (15.46%)	3266.9	515.1
**Low Birth Weight**					
No	419,920	376,816 (89.74%)	43,104 (10.26%)	3321.6	411.3
Yes	27041	7709 (28.51%)	19,332 (71.49%)	2182.7	312.1
**GINDEX**					
Intensive	27,956	23,865 (85.37%)	4091 (14.63%)	3284.2	504.6
Adequate	262,554	229,839 (87.54%)	32,715 (12.46%)	3274.5	480.8
Intermediate	120,595	101,608 (84.26%)	18,987 (15.74%)	3220.4	489.6
Inadequate	31,194	25,441 (81.56%)	5753 (18.44%)	3176.1	511.1
No prenatal care	6	2 (33.33%)	4 (66.67%)	2780	605
Missing	4656	3770 (80.97%)	886 (19.03%)	3189.2	527
**Total**	446,961	384,525 (86.03%)	62,436 (13.97%)		

**Table 2:** Descriptive statistics for gestational age and co-exposure variables.

Variable	N	Min	Mean	Max	SD
Gestational Age (weeks)	534,798	30.0	38.8	42.0	1.6
Ozone Trimester 1 (ppm)	458,130	0.02	0.04	0.08	0.01
Ozone Trimester 2 (ppm)	460,151	0.02	0.04	0.08	0.01
Ozone Trimester 3 (ppm)	460,239	0.02	0.05	0.09	0.01
PM_10_ Trimester 1 (µg/m^3^)	475,166	4.8	23.1	63.6	4.4
PM_10_ Trimester 2 (µg/m^3^)	474,702	4.8	23.0	60.9	4.3
PM_10_ Trimester 3 (µg/m^3^)	473,888	3.1	23.0	64.4	4.6
Non-Smoke PM_2.5_ Trimester 1 (µg/m^3^)	534,798	1.8	7.1	15.2	1.6
Non-Smoke PM_2.5_ Trimester 2 (µg/m^3^)	534,798	1.8	7.0	14.4	1.6
Non-Smoke PM_2.5_ Trimester 3 (µg/m^3^)	534,798	1.8	6.9	19.7	1.6
Wildfire Smoke PM_2.5_ Trimester 1 (µg/m^3^)	534,798	0.0	0.2	3.3	0.4
Wildfire Smoke PM_2.5_ Trimester 2 (µg/m^3^)	534,798	0.0	0.2	3.0	0.4
Wildfire Smoke PM_2.5_ Trimester 3 (µg/m^3^)	534,798	0.0	0.2	4.5	0.4
Temperature Deviation Trimester 1 (°F)	500,887	−44.0	0.1	18.5	2.2
Temperature Deviation Trimester 2 (°F)	502,681	−67.6	−0.1	20.1	2.1
Temperature Deviation Trimester 3 (°F)	503,105	−68.0	0.0	17.8	2.2

**Table 3:** Crude and adjusted associations between wildfire smoke PM_2.5_ (µg/m^3^) exposure and preterm births.

	Crude Model		Adjusted Model *	
	OR (95% CI)	*p*-Value	OR (95% CI)	*p*-Value
First Trimester	0.972 (0.950, 0.995)	0.019	1.024 (0.986, 1.065)	0.22
Second Trimester	1.058 (1.034, 1.082)	<0.0001	1.132 (1.088, 1.178)	<0.0001
Third Trimester	0.949 (0.930, 0.969)	<0.0001	1.013 (0.978, 1.050)	0.47
Mean Over All Trimesters	0.992 (0.980, 1.004)	0.19	1.055 (1.033, 1.078)	<0.0001
Full Gestation	0.972 (0.938, 1.006)	0.11	1.076 (1.016, 1.139)	0.013

* Model adjusted for: Ozone, non-wildfire PM_2.5_, PM_10_, temperature deviation, month, year, mother’s race/ethnicity, mother’s education and income, mother’s age, smoking during pregnancy, drinking during pregnancy, maternal asthma, and gindex.

**Table 4:** Crude and adjusted associations between wildfire smoke PM_2.5_ exposure (µg/m^3^) and birth weight (g).

	Crude Model		Adjusted Model *	
	Estimate (95%CI)	*p*-Value	Estimate (95% CI)	*p*-Value
First Trimester	2.9 (−1.1, 6.9)	0.16	−5.7 (−11.1, −0.4)	0.036
Second Trimester	11.7 (−15.7, −7.8)	<0.0001	3.0 (−2.7, 8.6)	0.3
Third Trimester	4.2 (0.8, 7.7)	0.017	−3.4 (−8.3, 1.6)	0.19
Mean Over All Trimesters	−1.5 (−3.6, 0.5)	0.14	−2.0 (−5.0, 0.9)	0.18
Full Gestation	−4.8 (−10.8, 1.1)	0.11	−2.0 (−9.9, 5.9)	0.61

* Model adjusted for: Ozone, non-wildfire PM_2.5_, PM_10_, temperature deviation, month, year, mother’s race/ethnicity, mother’s education, income, mother’s age, smoking during pregnancy, drinking during pregnancy, maternal asthma, gindex, and gestational age.

**Table 5:** Adjusted associations between wildfire smoke PM_2.5_ exposure (µg/m^3^) and secondary outcomes.

		Crude Association		Adjusted Association ^1^	
Outcome	Model/Parameter	OR (95% CI)	*p*-Value	OR (95%CI)	*p*-Value
Gestational Diabetes					
	Distributed Lag Model ^2^				
	First Trimester	1.037 (0.993, 1.082)	0.10	1.144 (1.064, 1.230)	0.0003
	Second Trimester	1.075 (1.032, 1.120)	0.0006	1.017 (0.943, 1.096)	0.66
	Third Trimester	1.109 (1.070, 1.150)	<0.0001	1.048 (0.982, 1.117)	0.16
	Mean	1.073 (1.050, 1.097)	<0.0001	1.068 (1.027, 1.111)	0.0012
	Full Gestation Model ^3^	1.238 (1.163, 1.317)	<0.0001	1.151 (1.034, 1.281)	0.010
Gestational Hypertension					
	Distributed Lag Model ^2^				
	First Trimester	1.215 (1.168, 1.264)	<0.0001	1.148 (1.071, 1.231)	0.0001
	Second Trimester	1.181 (1.135, 1.229)	<0.0001	1.124 (1.044, 1.211)	0.0020
	Third Trimester	1.071 (1.032, 1.111)	0.0003	0.959 (0.898, 1.024)	0.21
	Mean	1.154 (1.130, 1.178)	<0.0001	1.074 (1.033, 1.117)	0.0004
	Full Gestation Model ^3^	1.522 (1.434, 1.616)	<0.0001	1.204 (1.083, 1.339)	0.0006

^1^ Model adjusted for: Ozone, non-wildfire PM_2.5_, PM_10_, temperature deviation, calendar month, year, mother’s race/ethnicity, mother’s education, income, mother’s age, smoking during pregnancy, drinking during pregnancy, asthma, gestational age, and gindex. ^2^ Model includes trimester-specific parameters for wildfire smoke PM_2.5_, non-wildfire PM_2.5_, PM_10_, ozone, and temperature deviation. ^3^ Model includes a single, full gestation, parameter each for wildfire smoke PM_2.5_, non-wildfire PM_2.5_, PM_10_, ozone, and temperature deviation.

**Table 6:** Adjusted associations between wildfire smoke PM_2.5_ exposure (µg/m^3^) and secondary outcomes.

		Crude Association		Adjusted Association ^1^	
Outcome	Model/Parameter	OR (95%CI)	*p*-Value	OR (95%CI)	*p*-Value
NICU Admission					
	Distributed Lag Model ^2^				
	First Trimester	1.137 (1.102, 1.173)	<.0001	0.980 (0.923, 1.040)	0.50
	Second Trimester	1.142 (1.107, 1.177)	<0.0001	0.927 (0.871, 0.988)	0.019
	Third Trimester	1.074 (1.044, 1.104)	<0.0001	0.965 (0.913, 1.019)	0.20
	Mean	1.117 (1.099, 1.135)	<0.0001	0.957 (0.926, 0.989)	0.0093
	Full Gestation Model ^3^	1.371 (1.309, 1.436)	<0.0001	0.799 (0.730, 0.874)	<0.0001
Assisted Ventilation					
	Distributed Lag Model ^2^				
	First Trimester	0.891 (0.850, 0.934)	<0.0001	0.772 (0.712, 0.836)	<0.0001
	Second Trimester	0.871 (0.831, 0.914)	<0.0001	0.916 (0.843, 0.996)	0.041
	Third Trimester	0.827 (0.793, 0.863)	<0.0001	0.948 (0.881, 1.021)	0.16
	Mean	0.863 (0.841, 0.85)	<0.0001	0.875 (0.837, 0.915)	<0.0001
	Full Gestation Model ^3^	0.638 (0.592, 0.687)	<0.0001	0.583 (0.515, 0.659)	<0.0001

^1^ Model adjusted for: Ozone, non-wildfire PM_2.5_, PM_10_, temperature deviation, calendar month, year, mother’s race/ethnicity, mother’s education, income, mother’s age, smoking during pregnancy, drinking during pregnancy, asthma, gestational age, and gindex. ^2^ Model includes trimester-specific parameters for wildfire smoke PM_2.5_, non-wildfire PM_2.5_, PM_10_, ozone, and temperature deviation. ^3^ Model includes a single, full gestation, parameter each for wildfire smoke PM_2.5_, non-wildfire PM_2.5_, PM_10_, ozone, and temperature deviation.

**Table 7:** Adjusted associations between wildfire smoke PM_2.5_ exposure (µg/m^3^) and secondary outcomes.

		Crude Association		Adjusted Association ^1^	
Outcome	Model/Parameter	OR (95%CI)	*p*-Value	OR (95%CI)	*p*-Value
Small for Gestational Age					
	Distributed Lag Model ^2^				
	First Trimester	1.012 (0.977, 1.048)	0.49	1.060 (1.001, 1.124)	0.047
	Second Trimester	1.070 (1.034, 1.106)	<0.0001	1.040 (0.979, 1.104)	0.20
	Third Trimester	1.026 (0.995, 1.057)	0.098	1.023(0.971, 1.078)	0.39
	Mean	1.036 (1.017, 1.054)	0.0001	1.041 (1.008, 1.075)	0.013
	Full Gestation Model ^3^	1.121 (1.064, 1.180)	<0.0001	1.088 (0.999, 1.185)	0.054
Low Birth Weight (<2500 g)					
	Distributed Lag Model ^2^				
	First Trimester	1.011 (0.978, 1.045)	0.51	1.030 (0.962, 1.103)	0.40
	Second Trimester	1.076 (1.042, 1.111)	<0.0001	1.004 (0.936, 1.078)	0.90
	Third Trimester	0.983 (0.955, 1.012)	0.25	1.031 (0.971, 1.095)	0.31
	Mean	1.023 (1.005, 1.040)	0.011	1.022 (0.985, 1.060)	0.25
	Full Gestation Model ^3^	1.062 (1.011, 1.116)	0.017	1.075 (0.973, 1.187)	0.15

^1^ Model adjusted for: Ozone, non-wildfire PM_2.5_, PM_10_, temperature deviation, calendar month, year, mother’s race/ethnicity, mother’s education, income, mother’s age, smoking during pregnancy, drinking during pregnancy, asthma, gestational age, and gindex. ^2^ Model includes trimester-specific parameters for wildfire smoke PM_2.5_, non-wildfire PM_2.5_, PM_10_, ozone, and temperature deviation. ^3^ Model includes a single, full gestation, parameter each for wildfire smoke PM_2.5_, non-wildfire PM_2.5_, PM_10_, ozone, and temperature deviation.

## Supplementary Materials

The following are available online at https://www.mdpi.com/1660-4601/16/19/3720/s1, Figure S1: Map of the number of births in each ZIP code, 2007–2015, Figure S2: Map of the percentage of pre-term births in each ZIP code, 2007–2015, Figure S3: Map of mean birth weight in each ZIP code, 2007–2015, Table S1: Secondary Outcomes, Table S2: Sensitivity models for the association between wildfire smoke PM_2.5_ (μg/m^3^) exposure and preterm birth, Table S3: Sensitivity models for the association between wildfire smoke PM_2.5_ (μg/m^3^) exposure and birth weight (g).
