# Supplementary material for: Impact of Wildfire Smoke on Adverse Pregnancy Outcomes in Colorado, 2007–2015
Source: Int J Environ Res Public Health. 2019 Oct 2;16(19):3720. doi: 10.3390/ijerph16193720 (PMC6801422; doi:10.3390/ijerph16193720)
Supplement: Supplementary file 1 [file ijerph-16-03720-s001.pdf]

International Journal of
Environmental Research and Public Health

MDPI

Supplementary Materials

Impact of Wildfire Smoke on Adverse Pregnancy Outcomes in Colorado, 2007–2015

Mona Abdo, Isabella Ward, Katelyn O'Dell, Bonne Ford, Jeffrey R. Pierce, Emily V. Fischer and James L. Crooks *

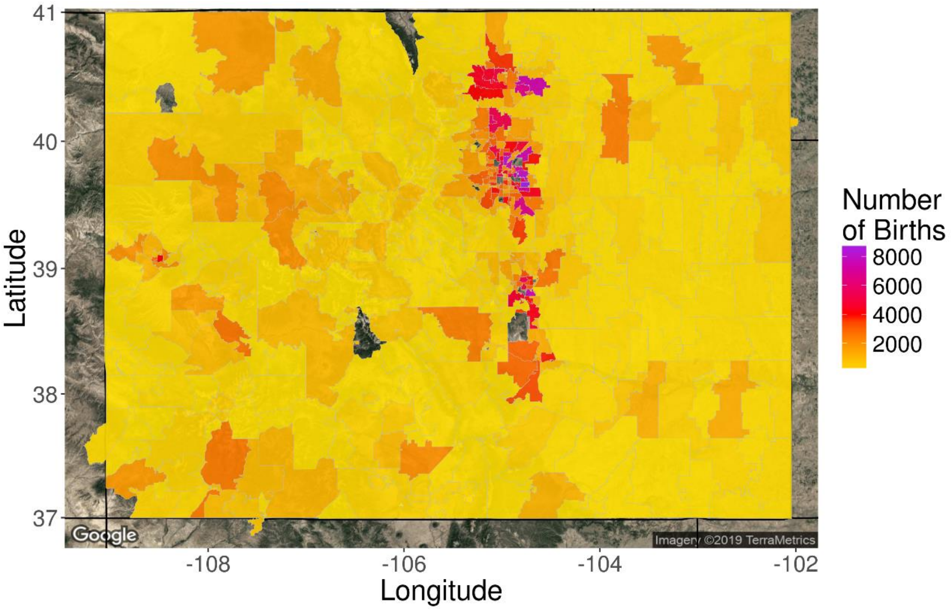

Figure S1. Map of the number of births in each ZIP code, 2007–2015.

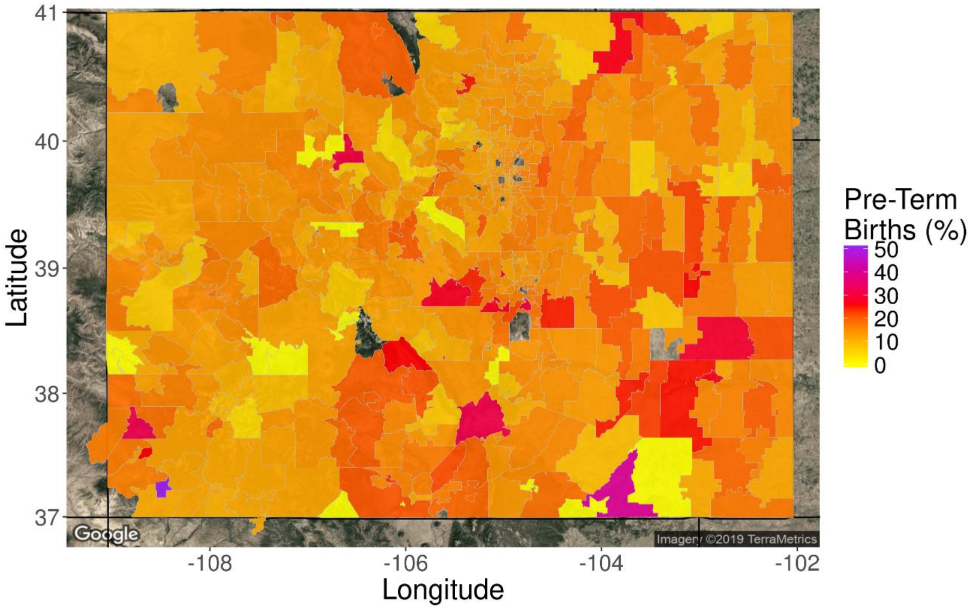

Figure S2. Map of the percentage of pre-term births in each ZIP code, 2007–2015.

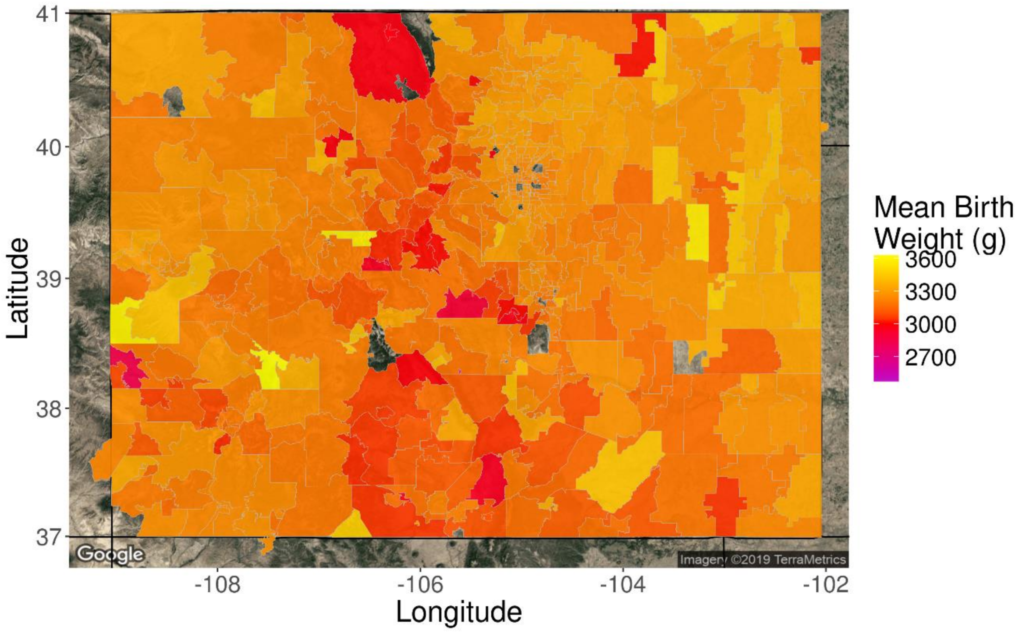

Figure S3. Map of mean birth weight (in grams) in each ZIP code, 2007–2015.

Table S1. Secondary Outcomes.

	Response		
**Secondary Outcome**	**N**	**No**	**Yes**
Gestational Diabetes	532747	512569	20178
Gestational Hypertension	530842	510775	20067
NICU Admission	535895	501855	34040
Assisted Ventilation	535895	515968	19927
Small for Gestational Age	535822	505115	30707
Low Birth Weight (<2500g)	535822	501694	34128

Table S2. Sensitivity models for the association between wildfire smoke $PM_{2.5}$ (μg/m³) exposure and preterm birth.

Sensitivity Models[1]	Model/Parameter	OR (95%CI)	P-value
**Main model without PM_{10}**			
	Distributed Lag Model[2]		
	First Trimester	0.995 (0.960,1.031)	0.78
	Second Trimester	1.128 (1.086,1.171)	<0.0001
	Third Trimester	0.988 (0.956,1.021)	0.47
	Mean	1.035 (1.016,1.054)	0.0003
	Full Gestation Model[3]	1.050 (0.998,1.105)	0.061
**Main model without air pollutants except wildfire smoke $PM_{2.5}$**			
	Distributed Lag Model[2]		
	First Trimester	0.99 (0.98,1.01)	0.79
	Second Trimester	1.12 (1.10,1.13)	<0.0001
	Third Trimester	0.96 (0.95,0.97)	<0.0001
	Mean	1.02 (1.01,1.03)	<0.0001
	Full Gestation Model[3]	1.018 (0.976,1.062)	0.41
**Main model with infant gender**			
	Distributed Lag Model[2]		
	First Trimester	1.024 (0.985,1.064)	0.23
	Second Trimester	1.132 (1.088,1.177)	<0.0001
	Third Trimester	1.013 (0.977,1.050)	0.48
	Mean	1.055 (1.033,1.078)	<0.0001
	Full Gestation Model[3]	1.074 (1.015,1.138)	0.014
**Main model without temperature**			

	Distributed Lag Model[2]		
	First Trimester	1.008 (0.972,1.046)	0.65
	Second Trimester	1.104 (1.063,1.146)	<0.0001
	Third Trimester	1.016 (0.983,1.051)	0.35
	Mean	1.04 2(1.022,1.062)	<0.0001
	Full Gestation Model[3]	1.078 (1.020,1.139)	0.0076
**Main model at 5 km buffer**			
	Distributed Lag Model[2]		
	First Trimester	0.930 (0.789,1.095)	0.38
	Second Trimester	1.225 (1.051,1.427)	0.0096
	Third Trimester	1.116(0.962,1.296)	0.15
	Mean	1.083 (0.991,1.184)	0.078
	Full Gestation Model[3]	1.130 (0.904,1.412)	0.28
**Main model at 10 km buffer**			
	Distributed Lag Model[2]		
	First Trimester	0.993 (0.932,1.057)	0.82
	Second Trimester	1.183 (1.109,1.262)	<0.0001
	Third Trimester	1.027 (0.968,1.089)	0.38
	Mean	1.064 (1.029,1.101)	0.0003
	Full Gestation Model[3]	1.096 (1.003,1.199)	0.047
**Main model at 50 km buffer**			
	Distributed Lag Model[2]		
	First Trimester	1.007 (0.972,1.043)	0.71
	Second Trimester	1.101 (1.061,1.142)	<0.0001
	Third Trimester	1.004 (0.971,1.038)	0.81
	Mean	1.036 (1.016,1.057)	0.0004
	Full Gestation Model[3]	1.057 (1.001,1.115)	0.045
**Main model without population weighting of wildfire smoke and non-smoke $PM_{2.5}$**			
	Distributed Lag Model[2]		
	First Trimester	1.024 (0.986,1.065)	0.22
	Second Trimester	1.133 (1.089,1.178)	<0.0001
	Third Trimester	1.014 (0.978,1.051)	0.47
	Mean	1.055 (1.033,1.078)	<0.0001
	Full Gestation Model[3]	1.076 (1.016,1.139)	0.012

[1]Main model adjusted for: Ozone, non-wildfire $PM_{2.5}$, PM_{10}, temperature deviation, calendar month, year, mother's race/ethnicity, mother's education and income, mother's age, smoking during pregnancy, drinking during pregnancy, asthma, and gindex. The main model used co-exposure data calculated using the 20 km buffer.
[2]Model includes trimester-specific parameters for wildfire smoke $PM_{2.5}$, non-wildfire $PM_{2.5}$, PM_{10}, ozone, and temperature deviation.
[3]Model includes a single, full gestation parameter each for wildfire smoke $PM_{2.5}$, non-wildfire $PM_{2.5}$, PM_{10}, ozone, and temperature deviation.

Table S3. Sensitivity models for the association between wildfire smoke $PM_{2.5}$ ($\mu g/m^3$) exposure and birth weight (g).

Sensitivity Models[1]	Model/Parameter	Estimate (95%CI)	P-value
**Main model without PM_{10}**			
	Distributed Lag Model[2]		
	First Trimester	-4.52 (-9.46, 0.43)	0.074
	Second Trimester	4.58 (-0.73, 9.88)	0.09
	Third Trimester	-0.59 (-5.17, 3.99)	0.80
	Mean	-0.18 (-2.74, 2.38)	0.89
	Full Gestation Model[3]	1.26 (-5.79, 8.31)	0.73
**Main model without air pollutants except wildfire smoke $PM_{2.5}$**			
	Distributed Lag Model[2]		
	First Trimester	-3.54 (-7.83, 0.75)	0.110
	Second Trimester	2.59 (-1.91,7 .08)	0.26
	Third Trimester	-1.16 (-4.94, 2.62)	0.55
	Mean	-0.71 (-2.71, 1.30)	0.49
	Full Gestation Model[3]	-0.92 (-6.86, 5.01)	0.76
**Main model with infant gender**			
	Distributed Lag Model[2]		
	First Trimester	-5.69 (-10.97, -0.41)	0.040

	Second Trimester	2.47 (-3.07, 8.01)	0.38
	Third Trimester	-3.61 (-8.51, 1.30)	0.15
	Mean	-2.28 (-5.19, 0.64)	0.13
	Full Gestation Model[3]	-2.70 (-10.51, 5.10)	0.50
**Main model without temperature**			
	Distributed Lag Model[2]		
	First Trimester	-4.57 (-9.65, 0.51)	0.080
	Second Trimester	5.26 (-0.04, 10.55)	0.050
	Third Trimester	-1.76 (-6.38, 2.86)	0.45
	Mean	-0.36 (-3.01, 2.29)	0.80
	Full Gestation Model[3]	0.024 (-7.56, 7.60)	0.99
**Main model at 5 km buffer**			
	Distributed Lag Model[2]		
	First Trimester	-7.03 (-29.18,1 5.13)	0.53
	Second Trimester	10.96 (-11.12, 33.03)	0.33
	Third Trimester	-3.63 (-24.68, 17.42)	0.74
	Mean	0.10 (-12.14, 12.34)	0.99
	Full Gestation Model[3]	2.76 (-28.04, 33.56)	0.86
**Main model at 10 km buffer**			
	Distributed Lag Model[2]		
	First Trimester	-2.88 (-11.51, 5.76)	0.51
	Second Trimester	4.10 (-5.04, 13.25)	0.38
	Third Trimester	-2.54 (-10.66, 5.57)	0.54
	Mean	-0.44 (-5.07, 4.19)	0.85
	Full Gestation Model[3]	2.21 (-10.05, 14.47)	0.72
**Main model at 50 km buffer**			
	Distributed Lag Model[2]		
	First Trimester	-3.88 (-8.77, 1.02)	0.120
	Second Trimester	2.64 (-2.61, 7.89)	0.32
	Third Trimester	-2.70 (-7.34, 1.94)	0.25
	Mean	-1.31 (-4.04, 1.41)	0.35
	Full Gestation Model[3]	-1.03 (-8.50, 6.44)	0.79
**Main model without population weighting of wildfire smoke and non-smoke $PM_{2.5}$**			
	Distributed Lag Model[2]		
	First Trimester	-5.92 (-11.27, -0.58)	0.03
	Second Trimester	3.08 (-2.54, 8.70)	0.28
	Third Trimester	-3.34 (-8.31, 1.64)	0.19
	Mean	-2.06 (-5.01, 0.89)	0.17
	Full Gestation Model[3]	-2.09 (-10.0, 5.83)	0.61

[1]Main model adjusted for: Ozone, non-wildfire $PM_{2.5}$, PM_{10}, temperature deviation, calendar month, year, mother's race/ethnicity, mother's education and income, mother's age, smoking during pregnancy, drinking during pregnancy, asthma, gindex, and gestational age. The main model used co-exposure data calculated using the 20 km buffer.

[2]Model includes trimester-specific parameters for wildfire smoke $PM_{2.5}$, non-wildfire $PM_{2.5}$, PM_{10}, ozone, and temperature deviation.

[3]Model includes a single, full gestation parameter each for wildfire smoke $PM_{2.5}$, non-wildfire $PM_{2.5}$, PM_{10}, ozone, and temperature deviation.

© 2019 by the authors. Submitted for possible open access publication under the terms and conditions of the Creative Commons Attribution (CC BY) license (http://creativecommons.org/licenses/by/4.0/).
